# The influence of adjunctive traditional Chinese medicine therapy on survival in primary liver cancer: a real-world study based on electronic medical records

**DOI:** 10.3389/fphar.2023.1231933

**Published:** 2023-09-18

**Authors:** Ruixia Zhao, Linlin Wang, Yibing Liu, Mingyi Shao, Wei Yang, Yu Fu, Qilong Gao, Jun’an Feng, Yunfei Xing, Xinghua Xiang

**Affiliations:** ^1^ The First Affiliated Hospital of Henan University of Chinese Medicine, Zhengzhou, Henan, China; ^2^ The First Clinical Medical College, Henan University of Traditional Chinese Medicine, Zhengzhou, Henan, China; ^3^ Institute of Basic Research in Clinical Medicine, China Academy of Chinese Medical Sciences, Beijing, China; ^4^ Department of Integrated Traditional Chinese Medicine and Modern Medicine, Henan Cancer Hospital, Zhengzhou, Henan, China; ^5^ Department of Digestive, People’s Hospital of Zhengzhou, Zhengzhou, Henan, China

**Keywords:** traditional Chinese medicine, primary liver cancer, real-world study, survival analysis, electronic medical record

## Abstract

**Background:** Traditional Chinese medicine (TCM) effectively improves the survival rate and quality of life of primary liver cancer patients, but high-level evidence is lacking.

**Patients and methods:** Patients were selected from 5 tertiary hospitals in Henan Province, China. Two thousand sixty-seven patients with primary liver cancer were included in the study. The electronic medical records (EMRs) of the patients were collected. Patients who received adjunctive TCM treatment and underwent treatment cumulative time for more than 1 month were classified as the TCM intervention cohort. Patients who did not receive adjunctive TCM treatment or underwent treatment cumulative time for less than 1 month were classified as the non-TCM intervention cohort. The main outcome indicators were the survival rate and overall survival time. The propensity score inverse probability weighting method was used to balance the differences between the groups.

**Results:** The primary cohort comprised 2,067 patients, including 462 patients who received adjunctive TCM treatment and 1,605 patients who did not receive adjunctive TCM treatment. The results of the Kaplan‒Meier survival curve indicated that the survival rate and median survival time of the exposure group before and after propensity score weighting were greater than those of the control group (*p* < 0.0001). Univariate Cox regression analysis after propensity score weighting showed that adjunctive TCM treatment was an independent protective factor for survival [regression coefficient = −0.215, hazard ratio (HR) = 0.8066, 95% confidence interval (CI) (0.6609–0.9844)].

**Conclusion:** Adjuvant treatment with TCM has a protective effect on the prognosis of patients with primary liver cancer; it can reduce the mortality and prolong the survival time.

## 1 Introduction

Liver cancer is predicted to be the fifth most important cancer worldwide, and it was the third leading cause of cancer death worldwide in 2020, with approximately 564,000 new cases and 549,000 deaths that year. Approximately 81% of cases occur in less developed countries, with 54% occurring in China (Zhou et al., 2019; Sung et al., 2021). Traditional Chinese medicine (TCM) is widely used in Chinese and East Asian societies, and the combination of TCM and conventional cancer treatment has a good effect in clinical practice. TCM treatments have targeted stimulation of the host immune response for cytotoxic activity against liver cancer by inhibiting proliferation and promoting the apoptosis of tumour cells (Cao et al., 2013; Ling et al., 2014a; Wu et al., 2014), thereby alleviating chemoradio therapy-related or gene therapy-related side effects (Konkimalla and Efferth, 2008; Ling et al., 2014b). However, these studies on TCM treatment for primary liver cancer have only been conducted in laboratories, with only one multicentre randomized control trial having evaluated the effects of TCM use in preventing recurrence after resection of small hepatocellular carcinoma (Zhai et al., 2013). To date, high-level evidence-based medicine evidence is still lacking.

For a long time, the clinical evaluation of TCM has remained at the empirical level, with related research results and clinical experience mostly presented in the form of case reports, which are considered the lowest level of evidence in evidence-based medicine, so the credibility of TCM is not optimal (Li et al., 2017). The higher the level of evidence in evidence-based medicine, the more conducive the findings are to guiding clinical practice. High-quality evidence is mostly found in randomized controlled trials, but this type of trial requires strict inclusion and exclusion criteria to eliminate nonspecific factors to the greatest extent possible. Although high-level clinical evidence has laid the foundation for the formulation of clinical practice guidelines for TCM, its extrapolation to actual medical practice has always been questionable (Liu et al., 2017).

With the rapid development of information technology and the advent of big data, high-tech approaches such as big data, artificial intelligence, internet, and cloud computing continue to emerge. The external environment of real-world research has undergone significant changes, and high-quality clinical big data are now easier to obtain. In addition, continuous improvements in research methods have made research results more reliable. Real-world studies have become an important supplement and continuation of randomized controlled trials (RCTs), and they are also a suitable method for evaluating curative effects within the context of the characteristics of TCM diagnosis and treatment (Fu et al., 2019; Fu et al., 2020). In recent years, hospital information systems at all levels have also been widely used and gradually improved. A patient’s complete medical records can be preserved, transmitted, managed and shared through an electronic medical record (EMR) system. Massive amounts of EMR data have laid a solid data foundation for knowledge discovery in the medical field (Zhao et al., 2019). Compared with other types of data, EMR data have the characteristics of a large data volume, objectivity, and convenient storage and transmission. Therefore, real-world research based on EMR data has gradually become a popular research topic.

This study was based on real-world clinical EMRs and used the generalized boosted model (GBM) propensity score weighting method to address the problems of nonrandom and confounding factors, which were found in real-world TCM clinical data. This study was performed with the aims of observing the efficacy of adjunctive TCM treatment in primary liver cancer patients and providing a practical basis for future real-world studies.

## 2 Materials and methods

### 2.1 Study design

A retrospective cohort study design was used in this study. Five tertiary hospitals in Henan Province were selected, including 3 hospitals that integrated Chinese and Western medicine and 2 hospitals that used Western medicine. Primary liver cancer patients who were hospitalized from 2015 to 2017 were selected. The patients were divided into an exposed group and a nonexposed group according to the application of TCM, and the survival condition of the patients was observed. The data were real-world clinical EMR data based mainly on the structured EMR system of the hospital, and they were extracted from the medical record homepage in the medical record system and from the hospitalization information. This study was approved by the Ethics Committee of The First Affiliated Hospital of Henan University of Chinese Medicine (No. 2017HL-077), written informed consent was not required for this study.

### 2.2 Patients

This study mainly included inpatients with primary liver cancer in 5 tertiary hospitals from 2015 to 2017. A total of 2,067 patients were enrolled. The inclusion criteria were as follows: ①patients diagnosed with primary liver cancer consistent with the “Guidelines for the Standardized Pathological Diagnosis of Primary Liver Cancer (2011 Edition)” (Bureau of Medical Administration, National Health and Family Planning Comission of the People’s Republic of China, 2017); ②patients aged ≥18 years old; ③patients with complete relevant examination results and hospitalization information; ④patients and their families who were willing to cooperate with follow-up visits and follow-up calls, the data from which were included in the collected information. The exclusion criteria were as follows: ①patients with malignant tumours of other sites or with serious organic diseases of the heart, kidney, and other organs; ②patients whose original data were severely insufficient; and ③patients who were only recorded for the first time and subsequently lost to follow-up.

### 2.3 Data source

The data of this study were based mainly on real-world clinical EMRs. Through data collection and data preprocessing, the information in the clinical EMRs was extracted, and the data were cleaned and standardized. Finally, a relatively standardized database was established. The patient information collected in the study included the medical records of patients with primary liver cancer admitted to 5 tertiary hospitals from 2015 to 2017, as well as the telephone follow-up information for patients from August 2018 to March 2019. The EMR information mainly included basic demographic information, disease-related information (family history, drinking history, history of illness), indicators related to disease progression (Child‒Pugh classification, Barcelona Clinic Liver Cancer (BCLC) staging, Chinese staging, complications, etc.), admission/discharge information, and disease treatment-related information (surgery treatment and Chinese medicine intervention). The patient follow-up information obtained by telephone follow-up mainly included the patient’s final outcome, time of diagnosis, time of death and information related to the patient’s out-of-hospital medication use.

### 2.4 Exposure

In this study, adjunctive TCM treatment was regarded as the exposure factor, and the cohort was divided according to the degree of adjunctive TCM treatment received by primary liver cancer patients. Based on former methods, TCM users were identified as those who had received TCM and treatment cumulative time for more than 1 month, whereas those treatment cumulative time for less than 1 month were considered to be non-TCM users. (Shi et al., 2013; Lin et al., 2015; Tsai et al., 2016; Li et al., 2019). TCM treatment included TCM decoction treatment, Chinese patent medicine treatment, and TCM characteristic therapies (acupuncture, massage, external treatment, etc.).

The collection of exposed drugs and time is divided into two parts: 1) during hospitalization: according to the prescribed dose and time of medication recorded in the hospital electronic medical record information; 2) After discharge: supplement according to the type of medication and medication time of the patient obtained by telephone follow-up.

### 2.5 Outcome variables

The main observation indicators in this study were the survival rate and overall survival time of primary liver cancer patients. The overall survival time of patients during the study period were recorded. The starting point of survival time was the time at which primary liver cancer was diagnosed, and the end point was the time when the patient died of liver cancer or the end of the study period.

### 2.6 Quality control

The data were exported from the “case registration system” by data engineers. The design of data extraction documents covers all the key information to be collected, and the extraction of data is carried out by designated professionals using the “manual double entry mode” entry to ensure the accuracy of data. A standardized dictionary was used to standardize data processing to ensure that the data governance process records were complete and traceable. Detailed follow-up contents and follow-up plans were determined. Follow-up data were obtained using the hospital follow-up system. Patients who failed to return to the clinic on time were followed up by telephone by professionals. There are inspection documents in each step of data verification, cleaning and transformation to avoid missing steps. The quality controller regularly monitors the data collection process and randomly selects the collection form to ensure the authenticity and integrity of the data.

### 2.7 Statistical analysis

According to the inclusion and exclusion criteria and the TCM exposure criteria, the data of patients exposed and not exposed to TCM were analyzed in this study. First, we statistically described the baseline conditions of all patients. Quantitative data were described by the mean ± standard deviation or median (upper and lower quartiles), and comparisons between groups were performed using a *t*-test or Wilcoxon rank sum test; qualitative data were analyzed using frequencies or percentages. Comparisons between groups were performed using the Chi-square test or Fisher’s exact test.

Screening for confounding factors was mainly based on the results of the comparison between the baseline groups, removing variables with *p* < 0.05, and combining the relevant literature and the recommendations of clinicians to choose individual variables. To balance confounding factors, the GBM propensity score weighting method was adopted. The weights were calculated by stable weighting method. The mean stabilized weight and the standard deviation of the stabilized weights were estimated to assess the validity of the positivity assumption. If the mean of the stabilized weights is far from one or if there are very extreme values, then this can be indicative of non-positivity or that the propensity score model has been misspecified. Otherwise, the positivity assumption is valid. In this study, the difference in propensity scores before and after weighting was represented by the Kolmogorov-Smirnov test statistic, Kolmogorov-Smirnov test is a useful non-parametric hypothesis test, which is mainly used to test whether a set of samples come from a certain probability distribution (one-sample K-S test). Or to see if two samples have the same distribution (two-sample K-S test). And a KS statistic <0.05 was regarded as equality between groups (Yang et al., 2017; Dai et al., 2020; Xu et al., 2020).

The Kaplan‒Meier method was used to calculate the survival rate and draw a survival curve, and the survival rate was compared between groups by the log-rank test. Cox proportional hazard regression analysis was performed to observe the influence of adjunctive TCM intervention on the survival outcome and survival time of primary liver cancer patients and related risk factors. The BCLC stage was used as a subgroup to analyze the effect of traditional Chinese medicine treatment on the survival rate and overall survival time of primary liver cancer in different subgroups.

Sensitivity analysis of potential confounding recognition: Propensity score weighting methods can adjust for observable variables, but not for unobserved factors, namely, potential bias. The presence of potential bias can lead to the phenomenon that individuals with the same observed values of the covariate have different treatment assignment probabilities, that is, treatment assignment depends on the unobserved covariate. Therefore, we needed to identify possible potential confounding factors; sensitivity analysis tests whether a model is sensitive to potential confounding bias by sequentially removing confounding variables from the model.

In addition, taking into account that unbalanced confounding factors remained after propensity score weighting, as well as clinical experience and the single-factor analysis results, the factors that had an important impact on the outcome were screened out: cancer thrombus and liver cirrhosis. We carried out a cox multivariate analysis after propensity score weighting, to further explore the impact of traditional Chinese medicine treatment on the survival of primary liver cancer, and used this as a sensitivity analysis. See the table in the annex.

## 3 Results

### 3.1 Study population

**FIGURE 1:**
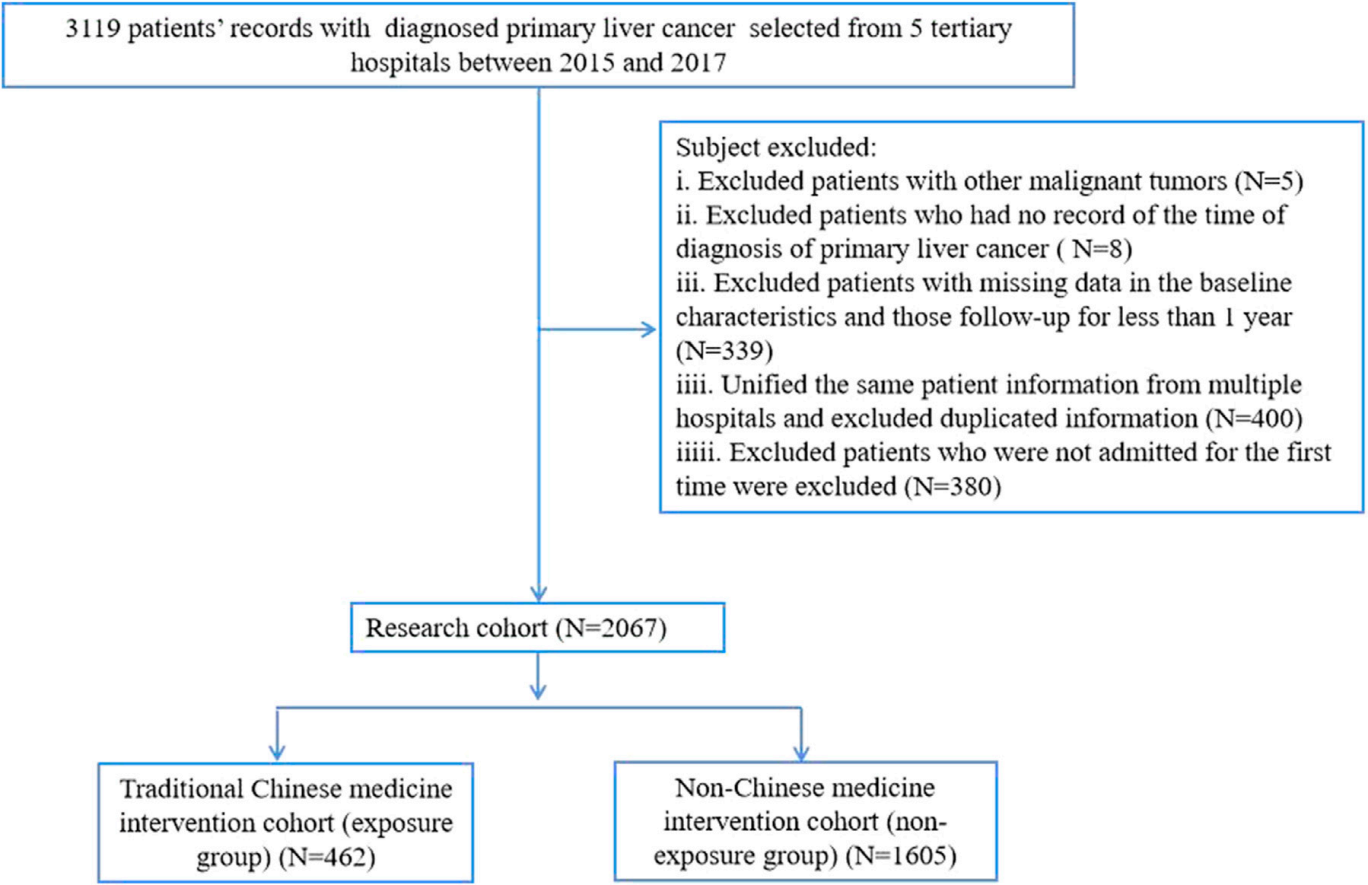
Chart of research cohort creation.

From 2015 to 2017, approximately 3,114 hospitalized patients were included from the 5 tertiary hospitals included in the study. Among them, 5 patients were excluded because they had other malignancies; 8 patients were excluded for lack of a time of diagnosis; 339 patients were excluded with missing data in the baseline characteristics and those follow-up for less than 1 year; During unified the same patient information from multiple hospitals, 400 patients were excluded for duplicated information; 380 patients were excluded because they were not admitted for the first time. Thus, 2,067 patients were ultimately included in the cohort, see as Figure 1.

### 3.2 Patient characteristics

Table 1 shows the baseline demographic characteristics and clinical characteristics of the study subjects before and after propensity score weighting. Among the 2,067 study subjects included, according to the exposure standards, 462 patients were treated with adjunctive TCM therapy; they had an average age of 57.51 years (standard deviation, 10.91), with 79% being male and 21% being female. A total of 1,605 patients did not use adjunctive TCM treatments; they had an average age of 56.23 (standard deviation 11.19) years, with 82% being male and 18% being female. Patients who used adjunctive TCM therapy were more likely to have the following characteristics: family history of liver cancer or viral hepatitis; history of hepatitis C, cirrhosis, or alcoholic liver disease; and complications of hepatic encephalopathy or abdominal infection. Patients treated with TCM assistance may have had higher BCLC staging or Chinese staging.

**TABLE 1:** Baseline demographic and clinical characteristics of patients before and after propensity score weighting.

	Unweighted			ks.pval	Propensity score weighted			ks.pval
Variable	Adjunctive TCM users	TCM nonusers	ks		Adjunctive TCM users	TCM nonusers	ks	
	*(N* = 462)	*(N* = 1,605)			(*N* = 462)	(*N* = 1,605)		
Age, mean (SD)	57.51 ± 10.95	56.23 ± 11.19	0.05	0.15	56.62 ± 11.07	56.35 ± 11.1	0.03	0.81
Sex, 100%								
Male	365 (79)	1316 (82)	0.02	0.28	370 (80)	1300 (81)	0.01	0.56
Female	97 (21)	289 (18)	0.02	0.28	92 (20)	305 (19)	0.01	0.56
Career, %								
Worker	11 (2)	32 (2)	0	0.74	11 (2)	32 (2)	0	0.86
Farmer	213 (46)	867 (54)	0.07	0	236 (51)	851 (53)	0.02	0.5
Leader	5 (1)	16 (1)	0	0.43	5 (1)	16 (1)	0	0.86
Teacher	5 (1)	16 (1)	0	0.47	5 (1)	16 (1)	0	0.69
Company employee	32 (7)	96 (6)	0.01	0.55	23 (5)	96 (6)	0.01	0.5
Other	134 (29)	417 (26)	0.03	0.23	134 (29)	433 (27)	0.02	0.38
Retiree	55 (12)	144 (9)	0.03	0.08	42 (9)	144 (9)	0	0.91
Medical insurance, %								
Provincial medical insurance	55 (12)	127 (8)	0.04	0.01	42 (9)	127 (8)	0.01	0.56
City medical insurance	46 (10)	127 (8)	0.02	0.19	37 (8)	127 (8)	0	0.89
NCMS^a^	259 (56)	963 (60)	0.04	0.17	273 (59)	963 (60)	0	1
None	92 (20)	337 (21)	0.02	0.38	92 (20)	337 (21)	0.01	0.58
Drinking history, %	148 (32)	562 (35)	0.03	0.27	166 (36)	562 (35)	0.01	0.8
Family history, %								
Liver cancer	55 (12)	144 (9)	0.03	0.05	51 (11)	144 (9)	0.02	0.41
Viral hepatitis	92 (20)	225 (14)	0.06	0.01	74 (16)	241 (15)	0.02	0.46
Medical history, %								
Hepatitis B	347 (75)	1220 (76)	0.01	0.58	347 (75)	1236 (77)	0.02	0.44
Hepatitis C	42 (9)	80 (5)	0.03	0	28 (6)	80 (5)	0.01	0.58
Liver cirrhosis	273 (59)	562 (35)	0.25	0	208 (45)	610 (38)	0.07	0.02
Alcoholic hepatitis	9 (2)	16 (1)	0.01	0.01	5 (1)	16 (1)	0.01	0.2
Complications, %								
Hepatic encephalopathy	28 (6)	48 (3)	0.04	0	18 (4)	48 (3)	0.01	0.26
Pulmonary infection	42 (9)	64 (4)	0.04	0	32 (7)	80 (5)	0.02	0.15
Upper gastrointestinal bleeding	18 (4)	64 (4)	0.01	0.58	14 (3)	64 (4)	0	0.61
Disease classification, %								
Child-Pugh stage								
A	259 (56)	819 (51)	0.05	0.09	259 (56)	835 (52)	0.05	0.1
B	106 (23)	385 (24)	0.01	0.64	97 (21)	401 (25)	0.03	0.19
C	65 (14)	273 (17)	0.04	0.08	60 (13)	273 (17)	0.03	0.15
BCLC stage								
0	9 (2)	32 (2)	0.01	0.32	14 (3)	32 (2)	0.01	0.23
A	102 (22)	385 (24)	0.01	0.53	106 (23)	385 (24)	0	0.83
B	134 (29)	353 (22)	0.07	0	120 (26)	353 (22)	0.03	0.16
C	116 (25)	514 (32)	0.06	0	129 (28)	498 (31)	0.03	0.26
D	65 (14)	241 (15)	0.01	0.65	65 (14)	225 (14)	0.01	0.66
Liver cancer stage								
Ⅰ	116 (25)	433 (27)	0.02	0.46	125 (27)	433 (27)	0	0.85
Ⅱ	125 (27)	305 (19)	0.08	0	102 (22)	321 (20)	0.02	0.41
Ⅲ	116 (25)	465 (29)	0.04	0.11	134 (29)	449 (28)	0	0.86
Ⅳ	65 (14)	289 (18)	0.04	0.02	60 (13)	289 (18)	0.04	0.06

The baseline treatment characteristics of patients before and after propensity score weighting are shown in Table 2. Some treatments, such as surgical excision, transcatheter arterial chemoembolization, hepatic artery embolism, radiofrequency ablation and microwave ablation, etc., were balanced after propensity score weighting.

**TABLE 2:** Baseline treatment characteristics of patients before and after propensity score weighting.

Variable	Unweighted			ks.pval	Propensity score weighted			ks.pval
	Adjunctive TCM users	TCM nonusers	ks		Adjunctive TCM users	TCM nonusers	ks	
	*(N* = 462)	*(N* = 1,605)			(*N* = 462)	(*N* = 1,605)		
Surgical treatment, %								
Surgical excision	129 (28)	610 (38)	0.09	0	162 (35)	594 (37)	0.02	0.53
Interventional therapy, %								
Transcatheter arterial chemoembolization	199 (43)	562 (35)	0.08	0	166 (36)	562 (35)	0	0.87
Hepatic artery infusion chemotherapy	92 (20)	337 (21)	0.01	0.62	88 (19)	337 (21)	0.02	0.39
Hepatic artery embolism	23 (5)	16 (1)	0.04	0	14 (3)	32 (2)	0.01	0.1
Local ablation, %								
Radiofrequency ablation	74 (16)	161 (10)	0.06	0	60 (13)	177 (11)	0.02	0.32
Microwave ablation	28 (6)	48 (3)	0.03	0	18 (4)	64 (4)	0.01	0.58
Antiviral therapy,%								
Entecavir	249 (54)	770 (46)	0.07	0.01	240 (52)	754 (47)	0.05	0.14
Lamivudine	46 (10)	80 (5)	0.05	0	32 (7)	80 (5)	0.02	0.16
Adefovir dipivoxil	65 (14)	128 (8)	0.07	0	55 (12)	128 (8)	0.03	0.08
Radiation therapy, %								
IODINE-125 implantation	42 (9)	112 (7)	0.02	0.24	32 (7)	112 (7)	0	0.85
Molecular targeting drug, %								
Sorafenib	23 (5)	96 (6)	0.01	0.4	28 (6)	96 (6)	0	0.94
Immunotherapy, %								
Interferon alph	9 (2)	0 (0)	0.02	0	5 (1)	16 (1)	0.01	0.16
Thymosin α1	69 (15)	161 (10)	0.05	0	65 (14)	161 (10)	0.03	0.06

**FIGURE 2:**
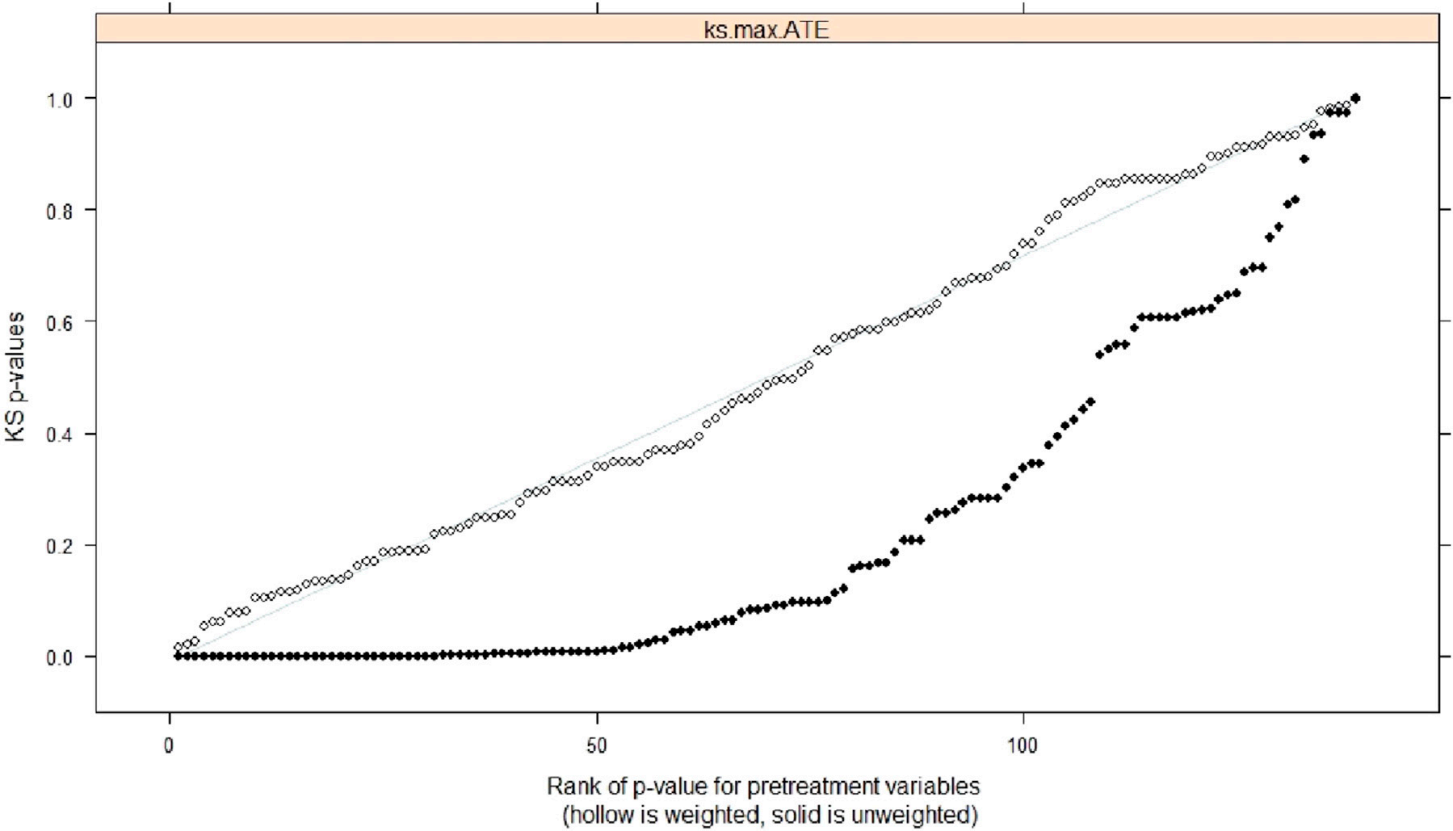
Comparison of the *p*-value and the uniform distribution value of the difference test between the two groups of confounding variables before and after weighting.

Figure 2 shows the *p* value and uniform distribution value of the confounding variables before and after weighting of the two groups. After propensity score weighting, the difference between the baseline confounding variables between the two groups was close to that expected with random assignment.

**FIGURE 3:**
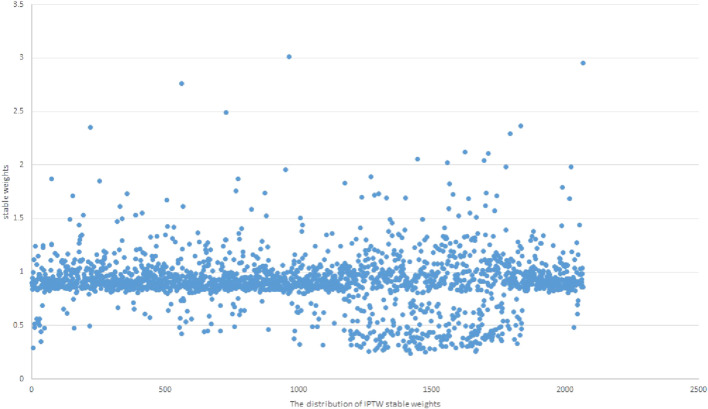
The distribution plot of the IPTW stable weights.

The mean stabilized weight and the standard deviation of the stabilized weights were estimated to assess the validity of the positivity assumption. If the mean of the stabilized weights is far from one or if there are very extreme values, then this can be indicative of non-positivity or that the propensity score model has been misspecified. Otherwise, the positivity assumption is valid. The distribution of the stable weights is shown in Figure 3, which has a mean of 0.91 and a standard deviation of 0.26, supporting the positivity hypothesis.

### 3.3 Primary outcomes

#### 3.3.1 Survival analysis

##### 3.3.1.1 Kaplan‒Meier analysis

**FIGURE 4:**
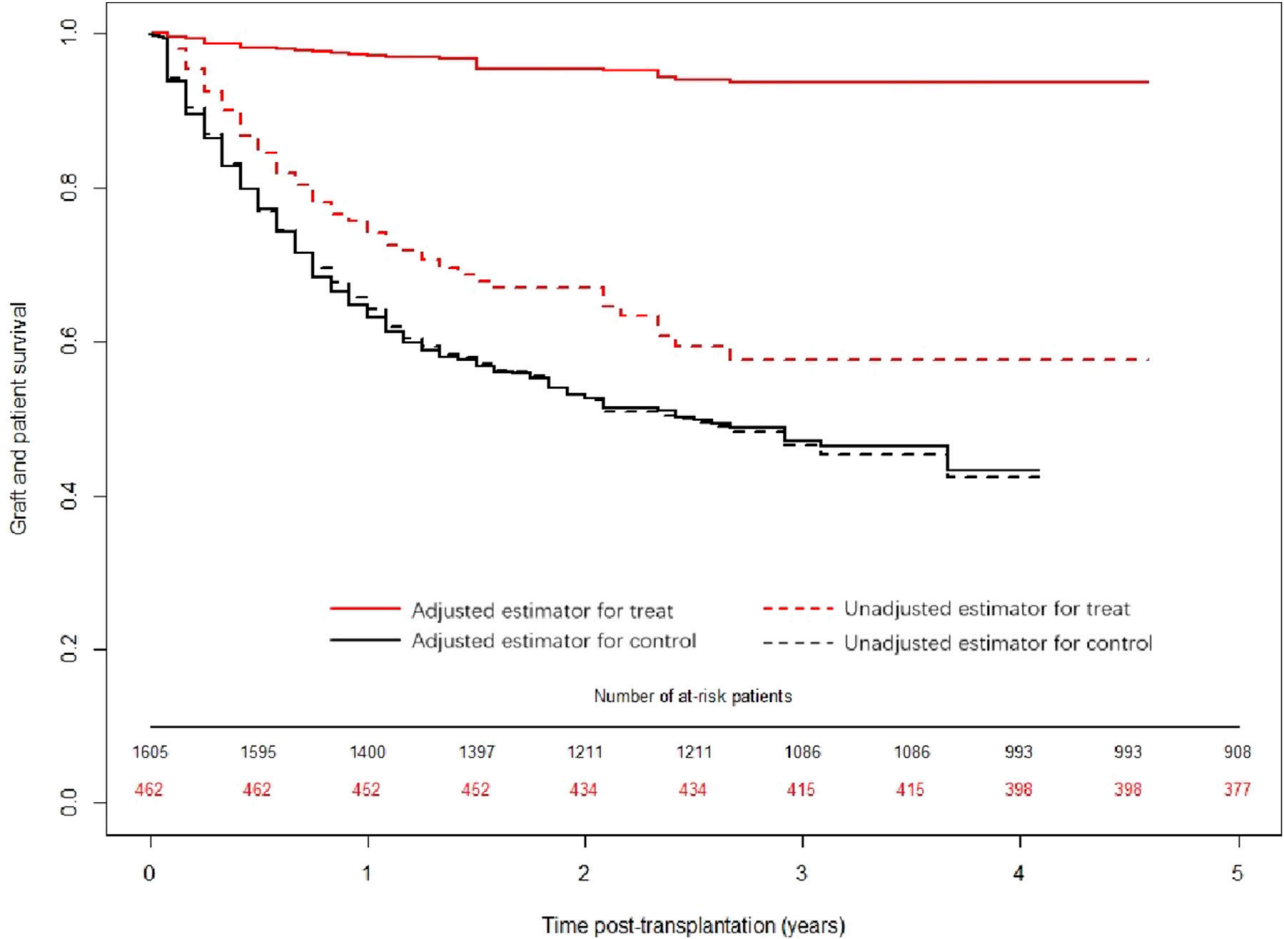
Distribution of the survival curves of the exposure group and control group before and after propensity score weighting.

Kaplan‒Meier analysis was used to calculate the survival rate and draw a survival curve, and the survival rate was compared between groups by the log-rank test, as shown in Figure 4. Before and after propensity score weighting, the median survival time of the TCM intervention group was longer than that of the control group, and the difference was statistically significant (*p* < 0.001).

##### 3.3.1.2 Cox regression analysis after the GBM trend score increased

The results of Cox univariate analysis before and after propensity score weighting are shown in the Table 3. The covariate-adjusted propensity score weighted Cox regression analysis showed that the regression coefficient for adjunctive TCM treatment was negative, and the difference was statistically significant (*p* = 0.0224). This suggests that adjuvant treatment with TCM has a protective effect on the prognosis of primary liver cancer patients; it can prolong the survival time and reduce mortality.

**TABLE 3:** Univariate Cox regression analysis before and after GBM propensity score weighting.

	Unweighted			Propensity score weighted		
Variable	Beta (SE)	HR (95% CI)	*P*	Beta (SE)	HR (95% CI)	*P*
Adjunctive TCM therapy	−0.4003	0.6701 (0.5561–0.8075)	<0.001	−0.215	0.8066 (0.6609–0.9844)	0.0344

#### 3.3.2 Supplementary analysis

##### 3.3.2.1 Hierarchical analysis--BCLC stage

**FIGURE 5:**
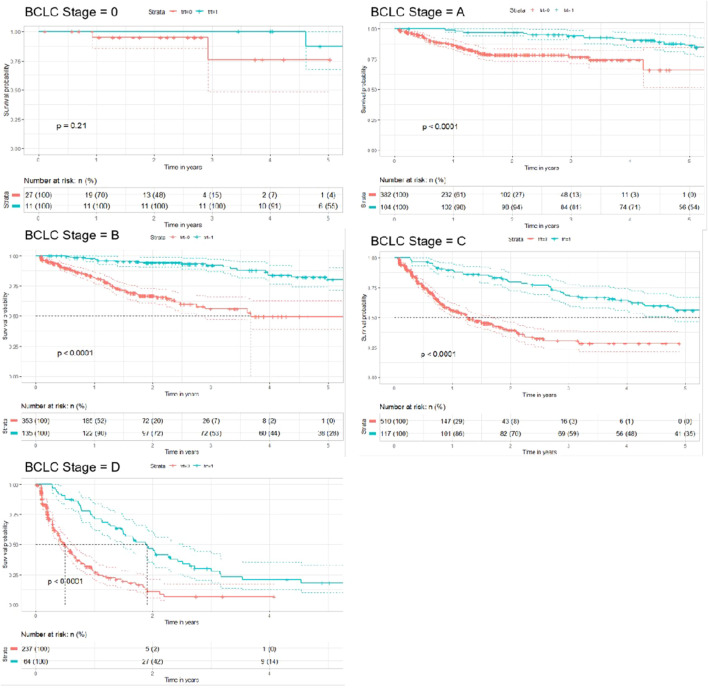
Survival curves after propensity score weighting for different stages of BCLC.

We used BCLC stage as a subgroup to explore the effect of TCM treatment on the prognosis of different stages of liver cancer. As shown in Figure 5, we plotted the survival curves of different stages of BCLC treated by propensity score weighting method and the results of Log-rank test, indicating that TCM treatment has a good effect on patients with different stages of BCLC of primary liver cancer.

##### 3.3.2.2 TCM treatment time

The treatment time of traditional Chinese medicine is also a key factor affecting the efficacy of traditional Chinese medicine. Previously, we divided the traditional Chinese medicine adjuvant treatment group and the non-traditional Chinese medicine adjuvant treatment group according to the treatment time. Here, we focused on the analysis of the relationship between the treatment time and the outcome of patients with primary liver cancer. We divided the duration of TCM treatment into four levels: level 1 for less than 3 months, level 2 for 3 months to 6 months, level 3 for 6 months to 1 year, and level 4 for more than 1 year. As shown in Table 4, a treatment duration of up to 6 months, 1 year, and even more than 1 year had a better effect compared with a treatment duration of less than 3 months.

**TABLE 4:** Association of TCM treatment duration with the prognosis of patients with primary liver cancer after GBM weighted.

Variable	Beta	HR (95% CI)	*P*
Time-to-treatment			
3 months ∼ 6months	−0.356	0.701 (0.446–1.101)	0.1226
6 months ∼ 12 months	−0.799	0.450 (0.269–0.751)	0.0023
>12 months	−1.357	0.257 (0.146–0.452)	<0.001

##### 3.3.2.3 Commonly used TCMs in patients with primary liver cancer

Commonly used TCMs in patients with primary liver cancer are shown in Table 5. The most common prescriptions of the TCM users were Hua Chan Su Jiao Nang, Fu Fang Ban Mao Jiao Nang, Yang Zheng Xiao Ji Jiao Nang, Ruan Gan Wan, Huaier Granule and Biejiajian Pills, which were used by 297 TCM users (29.6%), 172 TCM users (17.1%), 67 TCM users (6.7%), 63 TCM users (6.3%), 62 TCM users (6.2%) and 60 TCM users (6.0%) respectively (Table 5). Of the most common TCMs, 4 were herbal formulae, and 2 were single herbs.

**TABLE 5:** Commonly used TCMs in patients with primary liver cancer.

TCM name	Ingredients or generic name	Functional classification	No. of users	%
Cinobufagin Capsules	Toad Skin [Bufonidae; Dried toad skin]	Detoxicate disperse swelling and relieve pain	297	29.6
Compound Cantharidin Capsules	Mylabris [Meloidae; Mylabris phalerata Pallas]; Ginseng [Araliaceae; Ginseng Radix et Rhizoma]; Astragalus membranaceus [Leguminosae; Astragalus membranaceus Radix et Rhizoma]; Eleutherococcus senticosus [Araliaceae; Eleutherococcus senticosus Radix et Rhizoma]; Trigone [Sparganiaceae; Sparganium stoloni erum, Dry tubers of Sparganium stoloni erum]; Scutellaria barbata [Lamiaceae; Whole grass of Scutellaria barbata]; Curcuma phaeocaulis Valeton [ Zingiberaceae; Curcuma phaeocaulis Valeton Radix et Rhizoma]; Cornus officinalis [Cornaceae; Mature pulp of Cornus officinalis]; Ligustrum lucidum [Oleaceae; Mature fruit of Ligustrum lucidum]; Bear bile Powder [Ursidae; Dry Bile of Brown Bear and Black Bear]; Licorice [Leguminosae; Licorice Radix et Rhizoma];	Breaking blood stasis attack poison and corrode sore	172	17.1
Yang zheng Xiao ji Capsules	Astragalus membranaceus [ Leguminosae; Astragalus membranaceus Radix et Rhizoma ]; Ligustrum lucidum [Oleaceae; Mature fruit of Ligustrum lucidum]; Ginseng [Araliaceae; Ginseng Radix et Rhizoma]; Ganoderma lucidum [ Ganodermataceae; The fruiting body of Ganoderma lucidum]; Curcuma phaeocaulis Valeton [ Zingiberaceae; Curcuma phaeocaulis Valeton Radix et Rhizoma]; Atractylodes macrocephala [the composite family; Atractylodes macrocephala Radix et Rhizoma]; Hedyotis diffusa Willd [ Rubiaceae; The Whole Grass of Hedyotis diffusa Willd]; Scutellaria barbata [Lamiaceae; The Whole Grass of Scutellaria barbata]; Gynostemma pentaphyllum [ Cucurbitaceae; The Whole Grass of Gynostemma pentaphyllum]; Poria cocos [ Polyporaceae; Sclerotium of Poria cocos]; Gallusgallusdomesticus Brisson [ Phasianidae; Dry sac intima of chicken]; Duchesnea indica [Rosaceae; The Whole Grass of Duchesnea indica]; Solanum lyratum [ Solanaceae; The Whole Grass of Solanum lyratum]; Artemisia capillaris Thunb [the composite family; The Whole Grass of Artemisia capillaris]; Cynanchum paniculatum [Apocynum; Cynanchum paniculatum Radix et Rhizoma]; Eupolyphaga steleophaga [Periplanetidae; Female dried body of Eupolyphaga steleophaga]	Enforcing spleen and nourishing kidney, transform stasis and resolve toxin	67	6.7
Ruan Gan Wan	Carapax trionycis [Trionychidae; The back shell of turtle]; Carapax Testudinis [ Emydidae; Turtle belly armor]; Anis pentadactyla Linnaeus [ Manidae; The scales of Anis pentadactyla Linnaeus]; Angelica sinensis [Umbelliferae; Angelica sinensis Radix]; Oyster [Ostreidae; The shell of an oyster]; Peach kernel [Rosaceae; Dry mature seeds of Prunus persica]; Gallusgallusdomesticus Brisson [Phasianidae; Dry sac intima of chicken]; Licorice [Leguminosae; Licorice Radix et Rhizoma], Etc.	Activating blood circulation todissipate blood stasis, soften hardness and dissipate mass	63	6.3
Huaier granule	Sophora auricula mycoplasm [ Auriculariaceae; Fruiting body of Trametes robiniophila]	strengthen the body resistance to consolidate the constitution,·blood quickening and dissipate mass	62	6.2

## 4 Discussion

Adjunctive TCM treatment of primary liver cancer is widely used in China. Inpatients with liver cancer generally use TCM, such as TCM injections, for adjuvant treatment. The “Norms for the diagnosis and treatment of primary liver cancer (2019 edition)” pointed out that the treatment of liver cancer with traditional Chinese medicine can improve clinical symptoms, improve body resistance and quality of life, and reduce the adverse reactions caused by radiotherapy and chemotherapy. However, clinical studies on the therapeutic effects of TCM in patients with primary liver cancer remain scant, and to the best of our knowledge the majority are either case discussions or descriptive outcomes of a few patients. Although RCT studies are generally accepted as the gold standard, there are few RCT studies on TCM treatment of primary liver cancer, and some of the studies are not standardized and do not fully follow the principle of randomized control. In addition, this method has some limitations in the application of traditional Chinese medicine research, and its extrapolation is poor.

This study is a large-scale, real-world study based on clinical EMRs investigating the association between adjunctive TCM therapy and the survival of patients with primary liver cancer. More importantly, the GBM propensity score weighting method was used to balance the influence of confounding factors to achieve the “effect of postmortem randomization” and enhance the credibility of the evidence. The results of the study show that Chinese medicine interventions are independent protective factors in the survival of patients with primary liver cancer. The median survival time of the Chinese medicine-assisted intervention group was longer than that of the nonintervention group, and the survival rate was also improved. Our findings on the effects of TCM on the mortality of patients with primary liver cancer were similar to those of population-based studies. These studies used a retrospective cohort study to observe the effect of adjunctive TCM treatment on the survival of patients with advanced primary liver cancer, and the results all suggested that adjunctive TCM treatment can improve the survival rate of patients with primary liver cancer (Qiu et al., 2014; Liao et al., 2015), but these studies did not use appropriate methods to control confounding factors. In contrast with their studies, the GBM propensity score weighting method was adopted in this study, which controlled the confounding factors between the groups well, resulting in stronger evidence. In addition, some studies have shown that the use of Chinese medicine compounds in the treatment of primary liver cancer has a good effect (Shi et al., 2013; Li, 2016; Shi et al., 2016). A randomized controlled trial of TCM combined with transarterial chemoembolization (TACE) in the treatment of patients with unrespectable HCC showed that TCM can stimulate the host immune response by causing cytotoxic activity in liver cancer (Cho and Chen, 2009). In addition, some studies have shown that unit traditional Chinese medicine combined with targeted drugs has a remarkable effect on the treatment of HCC. As a zingiberaceae plant, the extract of Zedoary turmeric has the effect of inhibiting tumor growth, enhancing human immune system and reversing multiple drug resistance after the use of chemotherapy drugs. Chinese scholars have found that elemene injection combined with molecular targeted drugs can effectively improve the disease control rate and prolong the survival of patients with liver cancer (Sun and Zhang, 2012). In addition, as for the anti-tumor mechanism of traditional Chinese medicine, some studies have found that the immunomodulatory function of an approved Chinese medicine formula, compound kushen injection (CKI) acts on macrophages and CD8^+^ T cells to reshape the immune microenvironment of HCC, which improves the therapeutic outcomes of low-dose sorafenib and avoids adverse chemotherapy effects. It shows that traditional Chinese medicines with immunomodulatory properties can potentiate chemotherapeutic drugs and provide a promising approach for HCC treatment (Yang et al., 2020).

Several differences exist between the current study and previous population-based studies. First, the current study data were extracted from real-world clinical data based on electronic medical records with 2067 primary liver cancer patients who were more representative. Second, the electronic medical records included basic demographic information, disease information, laboratory examination information and treatment-related information, and it was possible to obtain all possible confounding factors, with further comprehensive control of these factors to reduce the occurrence of confounding bias. Third, the GBM propensity score weighting method was used to balance the influence of confounding factors, which can achieve the “effect of postmortem randomization” and enhance the credibility of the evidence.

This study also has some shortcomings. First, detailed patient discharge information, such as the type of medication, dosage, frequency, and duration of use, could not be accurately obtained. Electronic medical record data can only be used to collect information about patient medication use during hospitalization, not after discharge. The researcher’s prescribing frequency, the number of patients and the memories of patients or family members were used to infer the patient’s medication status at discharge. This causes a certain bias. Second, because the data were collected retrospectively, the patient’s disease was classified and staged. The information was stratified by the researchers based on the patient’s medical record information, making the results easily affected by the medical records. Besides, the inverse probability weighting method eliminates the influence of known covariates on the target effect, and the average efficacy of the various treatments obtained is comparable. In this case, the conclusion that TCM adjuvant therapy has a better effect is based on the average effect of the overall population, rather than on the individual. In addition, the inverse probability weighting method of propensity score was used in this study to control the confounding factors. This method can only control the known confounding factors, but cannot control the unknown confounding factors. However, in observational studies, there may be unknown confounding factors. Finally, in this study, the compositions of the Chinese medicine prescriptions that were taken were not recorded in detail, and the medication information could not be analysed in depth.

## 5 Conclusion

This study was based on real-world clinical EMRs and used the GBM propensity score weighting method to effectively control for nonrandom and confounding factors in the data; the effect of adjunctive TCM treatment on the survival of primary liver cancer patients was also explored. In this preliminary study, it was found that adjuvant traditional Chinese medicine therapy may improve the survival rate and prolong the survival time of patients with primary liver cancer. However, the specific effect of TCM interventions still needs to be further studied. This study provides a practical basis for future real-world research.

## Data Availability

The original contributions presented in the study are included in the article/Supplementary Material, further inquiries can be directed to the corresponding author.

## Abbreviations

AFP, alpha-fetoprotein; ASAM, average standardized absolute mean difference; BCLC, Barcelona Clinic Liver Cancer; CI, confidence interval; CT, computed tomography; DBIL, direct bilirubin; EMRs, electronic medical records; GBM, generalized boosted model; HR, hazard ratio; HCC, hepatocellular carcinoma; KS, Kolmogorov‒Smirnov; OR, odds ratio; P, probability; RCT, randomized controlled trial; SD, standard deviation; SE, standard error; TACE, transarterial chemoembolization; TCM, traditional Chinese medicine.
