# Supplementary material for: The influence of adjunctive traditional Chinese medicine therapy on survival in primary liver cancer: a real-world study based on electronic medical records
Source: Front Pharmacol. 2023 Sep 18;14:1231933. doi: 10.3389/fphar.2023.1231933 (PMC10544965; doi:10.3389/fphar.2023.1231933)
Supplement: Supplementary file 1 [file DataSheet1.PDF]

Attachments

Table 1. Cox regression with GBM propensity score weighted variables

Variable	Beta	HR (95% CI)	*P*
Adjunctive TCM therapy	-0.2477	0.7806 (0.6311-0.9655)	0.0224
Liver cirrhosis	0.17	0.8437 (0.6909-1.0303)	0.0954
Cancer embolus	1.0546	0.3483 (0.2831-0.4286)	<0.001
